# Good, bad, and neglectful: Astrocyte changes in neurodegenerative disease

**DOI:** 10.1016/j.freeradbiomed.2022.02.020

**Published:** 2022-03

**Authors:** Zoeb Jiwaji, Giles E. Hardingham

**Affiliations:** a UK Dementia Research Institute at the University of Edinburgh, Chancellor's Building, Edinburgh Medical School, EH16 4SB, UK; b Centre for Discovery Brain Sciences, University of Edinburgh, Hugh Robson Building, George Square, Edinburgh, EH8 9XD, UK

## Abstract

Astrocytes play key roles in CNS development as well as well as neuro-supportive roles in the mature brain including ionic, bioenergetic and redox homeostasis. Astrocytes undergo rapid changes following acute CNS insults such as stroke or traumatic brain injury, but are also profoundly altered in chronic neurodegenerative conditions such as Alzheimer's disease. While disease-altered astrocytes are often referred to as reactive, this does not represent a single cellular state or group of states, but a shift in astrocyte properties that is determined by the type of insult as well as spatio-temporal factors. Such changes can accelerate disease progression due to astrocytes neglecting their normal homeostatic neuro-supportive roles, as well as by gaining active neuro-toxic properties. However, other aspects of astrocytic responses to chronic disease can include the induction of adaptive-protective pathways. This is particularly the case when considering antioxidant defences, which can be up-regulated in many cell types, including astrocytes, in response to stresses, sometimes in concert with the activation of detoxification and proteostasis pathways. Protective responses, whilst potentially serving to mitigate neuronal dysfunction, may ultimately fail due to being insufficiently strong, or be offset by other deleterious changes to astrocytes occurring in parallel. Nevertheless, a greater understanding of early adaptive-protective responses of astrocytes to neurodegenerative disease pathology may point to ways in which these responses may be harnessed for therapeutic effect.

## Highlights

• Astrocytes play important homeostatic roles in the healthy brain.
• They are widely known to change for the worse in neurodegenerative disease.
• Changes include abandoning their homeostatic roles and becoming actively neurotoxic.
• Some responses however are protective and may be relevant to early stage disease.

## Graphical abstract

**Figure:**
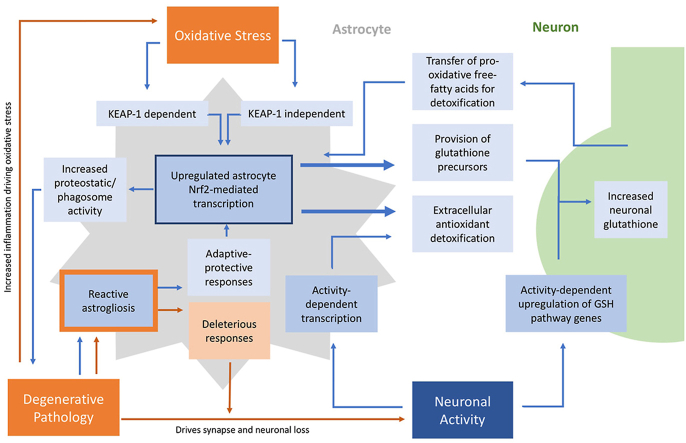

## 1 Introduction

Astrocytes, one of the most common CNS cell-types, play multiple roles essential for CNS development, homeostasis, and function (summarised in Table 1). However, their properties change during the course of neurodegenerative disease and models thereof. Whilst it has been known for over a hundred years that in response to disease astrocytes undergo morphological and histological changes (a term called reactive astrogliosis), recent studies have offered a deeper insight into the transcriptional, molecular and functional characteristics of this response [1]. These demonstrate that the astrocyte response in the degenerating brain is heterogenous in nature. Astrocytes can certainly acquire properties that are deleterious to CNS health (associated with a loss of homeostatic function or toxic gain of neurotoxic functions). Additionally, as in the case of antioxidant function, they may also demonstrate adaptive-protective responses which have the potential to confer neuroprotective benefit. In this review, we provide an overview of how astrocytes are well-adapted to provide antioxidant defences in the degenerating brain. Using selected examples from studies covering a range of different degenerative CNS diseases, we will describe how these responses change during disease and the outstanding challenges with regard to the manipulation of astrocytic function as a means of altering neurodegenerative disease trajectory.

**Table 1:** Key astrocyte functions.

Function	Description
**Synapse Formation and Neuronal Development**	
Synapse formation	Release of pro-synaptic molecules to increase synapse formation and function as part of the tripartite synapse [2].
Synapse elimination	Mediate synapse elimination through MEGF10 and MERTK pathways [3].
Neuronal growth	Secretion of neurotrophic growth factors (BDNF, NGF, GDNF) [4].
Secretion of extracellular matrix (ECM).	Express a range of proteoglycans essential for CNS extracellular matrix formation and neuronal adhesion molecules (*N*-cadherin, laminin and neural cell adhesion molecule (NCAM) during development, in health and following injury [5].
**CNS Homeostasis**	
Antioxidant function	Provide antioxidant support to nearby neurons (expanded below).
Glutamate uptake	Express glutamate transporters and play an essential role in CNS glutamate uptake and recycling [6].
Ammonia clearance	Detoxify ammonia by converting it into glutamine, with astrocyte dysfunction being implicated in hepatic encephalopathy [7].
Water homeostasis	Express aquaporin water channels on their basal membrane which are essential for CNS water homeostasis [8].
K^+^ balance	Elevated K^+^ following synaptic transmission is cleared by astrocytes; redistributed through astrocyte gap junctions and returned at sites of low K^+^ concentration via astrocyte Kir 4.1 channels [9].
**CNS Metabolism**	
Provision of metabolic precursors	Astrocytes are the main uptakers of CNS glucose from the blood, which has been proposed to be used to produce lactate for neuronal metabolic function as part of the astrocyte-neuron lactate shuttle [10,11].
CNS glycogen storage	Astrocytes are the predominant glycogen store in the CNS, and astrocyte glycogen is essential in protecting the brain from hypoglycaemia [12]
**Vascular Coupling**	
Vasomodulation and neurovascular modulation	Astrocytes contact the vasculature, and are hypothesised to be responsible for reactive hyperaemia – the process where blood flow in local parts of the brain is coupled to activity [13].
Regulation of blood brain barrier permeability	Astrocyte end-feet are one constituent of the BBB, and astrocyte transporter expression and end-feet anatomy can modulate BBB permeability [14].
**Injury Response**	
Formation of glial scar	Following injury, astrocytes become reactive and proliferate, forming a glial scar to contain inflammatory processes which can be beneficial for recovery [15].
Inflammatory cytokine production and complement activation.	Astrocytes secrete both proinflammatory and anti-inflammatory cytokines and chemokines, including Il-1, IL-6, TNF-alpha and IFN-gamma [16]. They also secrete complement factors C1q and C3, which activates complement and postulated to mediate synapse loss in dementia [17].
**Other Functions**	
Thyroid hormone activation	Uptake inactive T4 hormone from the blood and convert to active T3 [18]. Activated thyroid hormone is essential for myelination and brain development.
Cholesterol synthesis	Astrocytes have a key role in producing cholesterol. This is secreted and delivered to neurons as a complex with apolipoprotein (apo) E and required for neuronal membrane formation and synapse function [19].
Glymphatic flow	Astrocyte pulsatile motion is coupled with water egress through vascular-bound aquaporin channels required for pumping and clearing CNS waste through the glympathic system – the CNS equivalent of the lymphatic network [20]
Circadian rhythm	Astrocyte regulation of extracellular glutamate modulates the oscillatory patterns of neurons in the suprachiasmic nucleus to regulate night-time activity of the mammalian circadian clock [21].

## 2 Astrocytes play a key role in providing CNS antioxidant defence

Providing antioxidant support for the CNS in health and disease is a well-described function of astrocytes, and one for which they are well-adapted to. Astrocytes express high levels of nuclear factor-erythroid-derived 2-like 2 (Nrf2, gene name: Nfe2l2), a master regulator of antioxidant genes [22]. Under normal conditions, Nrf2 is bound to Kelch-like ECH-associated protein 1 (Keap1), allowing ubiquitination and targeting for proteosomal degradation [23]. In response to signals, including oxidative stress and heavy metal toxicity, the Nrf2-Keap1 interaction is disrupted, allowing an increase in intranuclear Nrf2 levels. Furthermore, in addition to the canonical Keap1-dependent Nrf2 activation pathway described, recent work has also demonstrated that oxidative-stress can activate Nrf2 in astrocytes via non-canonical Keap1-independent pathways [24]. Nrf2 promotes the transcription of a battery of genes regulated by the antioxidant-response element (ARE) sequence within their promoter sequences. This results in increased levels of genes whose products include antioxidant enzymes (e.g. catalase, peroxiredoxins, sulfiredoxin), antioxidant recycling and biosynthetic enzymes (e.g. glutathione reductase, thioredoxin reductase, gamma-glutamyl cysteine ligase) and detoxification enzymes (e.g. glutathione S-transferases, multi-drug-resistance-associated transporters) [25]. Interestingly, astrocytic Nrf2 also appears to have a degree of tonic activity that is important for neuronal and brain function, and that requires astrocytic ROS generation. Astrocytic oxidative phosphorylation leads to ROS generation via Complex I [26] and this mitochondrial ROS generation sustains a basal level of Nrf2 activity as well as reducing HDAC4 nuclear localization via its oxidation [27]. A consequence of this basal Nrf2 activity is to support glutathione production, and also to suppress NADPH oxidase (Nox) 1 and 2 [27]. HDAC4 nuclear exclusion de-represses miR-206, a negative regulator of glucose-6-phosphate dehydrogenase, the rate-determining step of the pentose phosphate pathway. Suppression of basal mitochondrial ROS production in astrocytes disrupts brain redox and metabolic homeostasis, leading to neuronal and cognitive dysfunction [27]. Of note, Keap1 knock-down has been reported to lead to elevated expression of Nox 4 and ROS production (potentially due an excessively reducing environment leading to increased nuclear HDAC4 [28]). Collectively this emphasizes the key role that astrocytic redox signaling plays in supporting neuronal metabolic states [11].

A role for astrocytes in providing redox support to neurons was established even before the discovery of Nrf2 as a major mediator [[29], [30], [31]]. Neurons have a relatively low intrinsic antioxidant capacity and do not store large amounts of glutathione [32,33]. This has been shown to be due in part to low Nrf2 expression as a result of epigenetic suppression of the gene early in development [34,35] and the low stability of what little Nrf2 is expressed at the protein level [36]. As such, Nrf2 does not contribute significantly to cortical intrinsic neuronal antioxidant defences, nor do neurons respond directly to pharmacological activators of Nrf2. Indeed, in the absence of a functional Nrf2 stress-response pathway, neurons may rely on activity-mediated Ca2+ dependent pathways to upregulate antioxidant genes rather than responding to redox changes [37,38]. However redox-mediated activation of Nrf2-driven transcription in astrocytes is able to confer neuroprotection via a mechanism that involves release and shuttling of astrocytic glutathione and glutathione precursors [39,40]. Glutathione, released from astrocytes in response to oxidative stress (via a mechanism that involves the multidrug resistance protein 1 (MRP1) transporter) [41], may assist in detoxifying the extra-cellular space. Neuronal activity also leads to transcriptional upregulation of genes for predominantly extracellular antioxidant molecules (Gpx 3 and Sod 3), suggesting the possibility that neurons control the ability of astrocytes to provide extracellular antioxidant capacity [42].

**Fig. 1:**
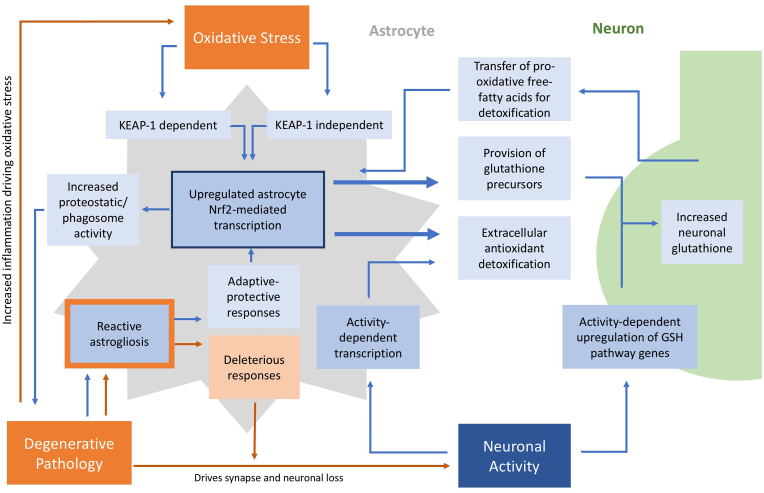
**Astrocyte-neuron interactions essential for CNS antioxidant homeostasis.** Pathways are modulated by both homeostatic mechanisms (neuronal activity) and pathological mechanisms (oxidative stress and degenerating pathology). Blue pathways are CNS-protective whilst orange pathways are those that worsen neurodegeneration.

Of note, recent studies have also demonstrated a role for astrocytes in protecting neurons from free-radical toxicity that may arise from detoxifying damaged mitochondrial membranes via mechanisms not directly linked to Nrf2 activation. This occurs both via transcellular transfer of mitochondria between neurons and astrocytes (transmitophagy) [43] a process by which healthy mitochondria protect neurons in a model of stroke [44]. Moreover, cytotoxic free-fatty acids produced as a result of degradation of damaged mitochondria are transferred from neurons (which have low intrinsic ß-oxidation pathways) to astrocytes via APOE positive lipid-droplets, where they can be metabolized by astrocyte mitochondrial beta-oxidation. This occurs along with appropriate astrocyte upregulation of antioxidant enzymic pathways to detoxify the resulting ROS generated from this process [45]. Fig. 1 summarizes the main interactions between neurons and astrocytes which influence redox homeostasis in the brain.

In addition to Nrf2-independent anti-oxidant/redox homeostasis function of astrocytes, it is important to note that Nrf2 activation can trigger cytoprotective functions that are not directly related to detoxifying ROS. Nrf2 is capable of regulating proteostasis, evidenced by a number of gain- and loss-of-function studies. Pharmacological activation of Nrf2 has been shown in to repress cytotoxicity and unfolded protein response (UPR) over-activation in response to ER stress inducers, via maintenance of ER redox balance and disulphide chemistry [[46], [47], [48]]. In addition to modulating the UPR, Nrf2 controls other arms of cellular proteostasis, such as the ubiquitin proteasome system (UPS) and the macroautophagy pathway [49,50].

## 3 Astrocytes react differently to different challenges

Astrocyte functions change significantly in response to disease, neurodegeneration and ageing. This phenomenon, called reactive astrogliosis (reviewed in detail recently [1]) was described over a century ago [51]. Reactive astrogliosis is a feature in both acute injury (stroke, acute inflammation, traumatic brain injury, spinal cord lesion) and chronic disease (AD, ALS, Parkinson's, Huntingdon's, MS), and is associated with a change in astrocyte morphology, altered Ca2+ signaling and transcriptomic changes [[52], [53], [54]]. The up-regulation of expression of the protein GFAP (glial fibrillary acidic protein) is a feature of most reactive astrocytes both in the context of acute and chronic disease states. As a marker of reactive astrocytes it is reliable, but potentially contributed to the view that GFAP-positive reactive astrocytes represented a single state. However, it is clear that reactive astrocytes cover a vast range of cellular states dependent on myriad factors including the nature and strength of the CNS insult, and the period of exposure [1]. An early illustration of this revealed that even the immediate transcriptional responses of astrocytes to different types of insult (LPS-induced neuroinflammation and transient ischemia), are distinct from one another, albeit with an element of overlap [55]. The picture in neurodegenerative disease is likely to be even more complex [1] with ‘reactive’ astrocyte properties depending on the specific disease, as well as disease stage and also brain region or proximity to other pathological features (such as ß-amyloid plaques).

## 4 Neurodegeneration can result in a deleterious loss of homeostatic functions in astrocytes

Changes to astrocytes in neurodegenerative disease can accelerate disease progression in two distinct ways. The first is through the decline of astrocytes’ ability to carry out the type of homeostatic functions described above in Table 1 [56]. Whilst a detailed description of these changes is beyond the scope of this review, important consequences of neurodegenerative processes include dysregulation of glutamate uptake recycling and impaired metabolic capacity. For example, astrocytes in AD have reduced glutamate uptake capacity and transporter expression [57], as well as exhibiting direct ROS-induced reduction in glutamate-transport capacity. Single-nucleus analysis of AD post-mortem tissue, along with analysis of astrocytes in AD mouse models, revealed transcriptomic changes consistent with metabolic and mitochondrial dysfunction, reduced neurotransmitter uptake capacity and impaired cholesterol cycling [[58], [59], [60], [61]]. These changes may impact on CNS antioxidant capacity in several ways. For example, reduced astrocytic glucose metabolism may limit glutathione recycling, reliant on NADPH produced from glucose metabolized through the pentose phosphate pathway. Other potential routes include loss of glutamate homeostasis leading to excessive Ca2+ influx into neurons, increasing metabolic demand, driving mitochondrial dysfunction and increased generation of ROS. The second potential way by which astrocytes can have deleterious effects is through specific toxic gains of function. In response to acute inflammatory insults such as IFN-gamma, IL-1 and LPS astrocytes have detrimental effects on neuronal health via mechanisms that include upregulation of iNOS and production of NO and reactive oxygen species [[62], [63], [64]] as well as directly secreting inflammatory mediators. Recent work has also demonstrated that systemic inflammation drives the emergence of neurotoxic astrocytes via microglial-derived factors, which induces neurotoxicity through the production of toxic free fatty-acids and long-chain saturated lipids [65]. It will be of interest to determine whether such mechanisms also occur in chronic neurodegenerative diseases in humans, as toxic lipid production would represent a novel pathway to target. Moreover, it remains unclear precisely what aspects of the aforementioned astrocytic phenotypic changes contribute to neurodegenerative disease, though it is likely to be disease and stage-specific. Nevertheless, approaches designed to inhibit upstream signals mediating reactive astrocytic responses to neurodegenerative disease pathology are demonstrably protective in such models. Such astrocytic signals include deregulated Ca^2+^ signaling, the Ca^2+^-dependent transcription factor NFAT, pro-inflammatory JAK/STAT signaling, and the stress/inflammation responsive transcription factor NF-kB [[66], [67], [68], [69], [70]].

Further evidence for the role of deleterious astrocytes in driving degenerative disease comes from models of amyotrophic lateral sclerosis (ALS), where the trigger is not the astrocyte's response to external brain pathology, but the cell-autonomous effect of mutation-containing astrocytes. Free radical damage and antioxidant stress are implicated as key mechanisms underlying ALS, with one of the first mutations identified associated with familial ALS being mutations in the SOD1 gene [71] with a recent licenced drug being a free-radical scavenger (edaravone). However, it has emerged that besides SOD1 deficiency in motor neurons driving pathology via direct cell-autonomous means, SOD1 mutations in astrocytes induce detrimental non-cell autonomous effects. Mutant SOD1 expressing astrocytes cause neuronal death in both *in vitro* studies (using both rodent and human cells) [[72], [73], [74]], and *in vivo* as demonstrated by the ability of transplanted mutant SOD1 astrocytes to increase spinal MN death in wild-type animals. Finally, to support that the effects of astrocyte SOD1 mutations on neuronal pathology may not be due to simple loss of antioxidant function but rather a gain of toxic function, conditioned media from SOD1 mutant astrocytes is sufficient to drive neuronal death [75,75], via a mechanism that induces necroptosis in neurons [76]. Moreover, non-cell-autonomous neuro-toxicity induced by ALS mutation-harbouring astrocytes is not restricted to SOD1. Recently C9orf72 human iPSC-derived astrocytes harboring the ALS/FTD-causing C9orf72 expansion were shown to cause motor neuron deficits [77] which may form part of a number of deleterious consequences for the brain of glia containing this mutation [78].

## 5 An adaptive-protective astrocyte antioxidant response is a feature in the degenerating brain- a response that is “too little, too late”?

In addition to neurodegeneration inducing changes to astrocytes that are associated with deleterious effects as detailed above, there is also emerging evidence that reactive astrocytes have the potential to mount adaptive-protective responses to pathology which may delay patho-progression [79]. Many cell types respond to adverse conditions such as hypoxia, metabolic stress or temperature shock with measures designed to limit harm. This is particularly relevant with regard to astrocyte antioxidant defence pathways, activated by oxidative stress by the inhibition of Nrf2's negative regulator Keap1, and which may contribute to adaptive-protective responses to mild preconditioning insults such as brief ischemia [39,[80], [81], [82]]. Moreover, this pathway appears to be activated in chronic disease as well: the Nrf2 target gene NQO1 is upregulated in plaque-surrounding astrocytes in human AD post-mortem samples, but not in areas unaffected by pathology [83] and HMOX1 was found to be elevated in temporal cortex and hippocampus in patients with both AD and mild cognitive impairment [84], with elevation in early disease suggesting that Nrf2 activation occurs relatively early in human neurodegenerative processes rather than representing an end-stage process. While these studies provide evidence that the brain is experiencing a degree of oxidative stress, these markers represent a protective response to this stress, in contrast to other markers (such as lipid peroxidation or DNA oxidation) that may better reflect actual oxidative damage to brain tissue.

In our recent study of astrocyte-specific translatome changes, we investigated the separate astrocyte response to both ß-amyloidopathy and tauopathy using relevant mouse models (APP/PS1 and MAPT^P301S^ respectively) and sequencing mRNAs associated with tagged ribosomes expressed specifically in astrocytes [85]. We found that both AD-relevant pathologies induced changes that ordinarily occur in the astrocytes of old mice and had distinct but overlapping signatures which were enriched in genes known to change in astrocytes in human AD. Of note, while Aß and tau pathology both induced inflammatory pathways and showed deficits in energy metabolism, other responses were consistent with an adaptive protective response, including antioxidant and proteostasis pathways. Moreover, analysis of genes induced in both models showed enrichment in Nrf2 target genes, as defined by genes identified using published Nrf2 ChIP-seq data, as was well genes induced in sorted astrocytes from mice that over-express Nrf2 specifically in astrocytes (GFAP-Nrf2 mice). Immunohistochemistry revealed that classical Nrf2 target gene heme oxygenase 1 was induced in both models, and astrocyte TRAP-seq data revealed Nrf2 target genes induced in one or both models (including NAD(P)H dehydrogenase quinone 1, sequestosome-1, cathepsin B, microsomal glutathione S-transferase 3, glutathione synthetase, fatty acyl CoA reductase 2, biliverdin reductase A, aldo-keto reductase family 1, member C18, sulfiredoxin, peroxiredoxin 6, angiopoietin-1, catalase, thioredoxin reductase 1, glutathione reductase and glutathione peroxidase 1). Of note, crossing the GFAP-Nrf2 mouse onto the models of ß-amyloidopathy and tauopathy reduced pathological and functional deficits in these mice [85]. Collectively this suggests that activation of endogenous Nrf2 in astrocytes in response to Aß or tau pathology is indeed a protective response, potentially acting via a combination of antioxidant, detoxification and proteostatic effects. However it is clear that this response is insufficient (either too little, too late or both) to prevent patho-progression. Whether activation of endogenous Nrf2 in astrocytes alters disease trajectory would require models of Aß and tau pathology to be crossed onto mice with astrocyte-specific deletion of Nrf2. Nevertheless, pre-emptive activation of this pathway by genetic or pharmacological means clearly has non-cell autonomous neuroprotective effects in models of Aß and tau pathology, as it does in models of Parkinson's disease, ALS and cerebral hypoperfusion, and oxidative stress, in animal models and human cells [29,[86], [87], [88], [89], [90], [91]]. The benefits observed of enhancing astrocyte Nrf2 responses in a number of neurodegenerative conditions associated with proteinopathy may in part be due to a wider protective role of Nrf2 in promoting CNS clearance of misfolded protein, extending beyond that of acting as solely an enhancer of antioxidant responses.

## 6 Targeting glial antioxidant pathways for therapeutic benefit

Given the above body of evidence supporting that astrocytes exhibit a protective Nrf2 response in the degenerating brain there is interest in using pharmacological activation of Nrf2 in this context [92]. Considering the weak activity of the neuronal Nrf2 pathway due to the epigenetic suppression of the Nrf2 gene, it is likely that Nrf2 activators, without additional prior epigenetic de-repression [34], will modulate CNS antioxidant defences through enhancing glial rather than neuronal antioxidant capacity. Whilst a detailed discussion of these approaches are beyond the scope of this review, it is important to note that not all electrophilic Nrf2 activators (which themselves drive significant oxidative neurotoxicity in degenerative disease) will translate to clinical benefit. There is increasing interest in the potential of less toxic triterpenoid-class of Nrf2-activators such as RTA-404 (2-Cyano-3,12-dioxool-eana-1,9 (11)-dien-28-oyl] trifluoroethylamide (CDDO^TFEA^)) or the closely related RTA-408 (Omaveloxolone), which has recently been demonstrated to improve neurological function compared to placebo in Friedreich ataxia [93]. Finally, as discussed above, recent evidence suggests that an aspect of astrocytic Nrf2 activation occurs via mechanisms independent of the canonical KEAP-1 pathway [24]. Therefore, even in the degenerating brain, (where ROS-mediated stress may already have maximised KEAP-1 mediated Nrf2 activation) there remains the potential for pharmacological therapies to exploit KEAP-1 independent activation of Nrf2 to further enhance this astrocyte cytoprotective response.

## 7 Conclusion

A failure of antioxidant defence and CNS redox homeostasis is considered to be an important final common pathway driving neuronal death in many neurodegenerative diseases. Neurodegenerative processes induce profound reactive changes to astrocytes. Whilst it is clear that reactivity does ultimately lead to a detrimental failure in many key astrocyte functions such as glutamate homeostasis or metabolic function, we propose that the evidence supports that reactive astrocytes actually undergo adaptive-protective changes in the context of antioxidant support. However, whilst these changes have neuroprotective potential, they may be “too little, too late” to avert neuronal dysfunction and death in the context of disease. A fuller understanding of the changes that astrocytes undergo in chronic disease will point to therapies that prevent neuro-toxic changes, rescue deficits in homeostatic support, and facilitate (or boost) adaptive-protective pathways.
